# Tissue-based metabolomics reveals potential biomarkers for cervical carcinoma and HPV infection

**DOI:** 10.17305/bjbms.2019.4359

**Published:** 2020-02

**Authors:** Abulizi Abudula, Nuermanguli Rouzi, Lixiu Xu, Yun Yang, Axiangu Hasimu

**Affiliations:** 1 Department of Labour and Environmental Hygienics, School of Public Health, Xinjiang Medical University, Urumqi, China; 2 Department of Pathology, School of Basic Medicine, Xinjiang Medical University, Urumqi, China

**Keywords:** Cervical carcinoma, metabolomics, tumor tissue, biomarkers

## Abstract

Aberrant metabolic regulation has been observed in human cancers, but the corresponding regulation in human papillomavirus (HPV) infection-associated cervical cancer is not well understood. Here, we explored potential biomarkers for the early prediction of cervical carcinoma based on the metabolic profile of uterine cervical tissue specimens that were positive for HPV16 infection. Fifty-two fresh cervical tissues were collected from women confirmed to have cervical squamous cell carcinoma (SCC; n = 21) or cervical intraepithelial neoplasia (CIN) stages II-III (n = 20). Eleven healthy women constituted the controls (negative controls [NCs]). Real-time polymerase chain reaction (PCR) was performed to detect HPV infection in the tissues. High-resolution magic angle spinning nuclear magnetic resonance was utilized for the analysis of the metabolic profile in the tissues. The expression of rate-limiting enzymes involved in key metabolic pathways was detected by reverse-transcription quantitative PCR. An independent immunohistochemical analysis was performed using 123 cases of paraffin-embedded cervical specimens. A profile of 17 small molecular metabolites that showed differential expression in HPV16-positive cervical SCC or CIN II-III compared with HPV-negative NC group was identified. According to the profile, the levels of α- and β-glucose decreased, those of lactate and low-density lipoproteins increased, and the expression of multiple amino acids was altered. Significantly increased transcript and protein levels of glycogen synthase kinase 3 beta (GSK3β) and glutamate decarboxylase 1 (GAD1) and decreased transcript and protein levels of pyruvate kinase muscle isozyme 2 (PKM2) and carnitine palmitoyltransferase 1A (CPT1A) were observed in the patient group (*p* < 0.05). HPV infection and cervical carcinogenesis drive metabolic modifications that might be associated with the aberrant regulation of enzymes related to metabolic pathways.

## INTRODUCTION

Cervical squamous cell carcinoma (SCC) ranks the highest among all death-causing cancers among women; thus, it constitutes an economic burden on the population in developing countries [1,2]. Although early detection might improve the clinical outcome of cervical cancer, most cases have progressed to advanced stages at the time of diagnosis due to technical limitations [3]. Infection with human papillomavirus (HPV), particularly high-risk (HR) HPV (e.g. HPV16 or 18), is an important factor in SCC progression [4,5]. Positive HR HPV infection has been reported in all SCC patients with cervical cancer and most patients with precancerous lesions (cervical intraepithelial neoplasia [CIN] II-III) [6]. However, because of the long latency of tumorigenesis after HPV infection, HPV screening alone might not be sufficient for cancer prediction [7]. Several tumor biomarkers, such as p16, Ki-67 or p16/Ki-67 dual staining, are used for early cervical cancer detection, but these markers have very limited specificity because their regulation might not be dependent on HPV infection [8-10]. Therefore, early detection of SCC requires the identification of novel biomarkers with auxiliary diagnostic potential for HPV testing.

Rapid proliferation is a driving force used by malignant cells to accommodate the massive energy requirement of metabolic modifications or ‘reprogramming’. Most solid tumors exhibit the typical Warburg effect even under physiological oxygen conditions [11]. Accordingly, decreased tissue levels of glucose and ribulose in head and neck cancers are considered important tissue-based tumor markers [12]. In addition, increased ethanolamine levels in tumor tissues are correlated with worse survival in pancreatic adenocarcinoma [13], and differences in amino acid profiles have been observed between estrogen receptor-positive and estrogen receptor-negative breast cancer [14]. Lipid metabolism exhibits shifts during colorectal cancer progression from early to advanced stages and in a transgenic animal model of hepatic cellular cancer induced by hepatitis B virus X infection [15,16]. Alterations in choline and phospholipid metabolism have been documented in tumor tissues and patient-derived xenografts of breast cancer [17,18]. In contrast, prostate cancer cells shift their energy metabolism by oxidizing citrate and utilizing the Krebs cycle for oxidative phosphorylation; thus, these cells do not exhibit the Warburg effect [19]. Based on these findings, aberrant metabolic regulation in cancer cells is a common signature of tumorigenesis, and although partial modifications of distinct metabolic pathways have been characterized in various types of tumors, to the best of our knowledge, the modifications in cervical cancer have not been systematically studied.

Therefore, this study aimed to identify biomarkers with an auxiliary diagnostic potential for HPV-based prediction of cervical carcinoma. To achieve this goal, we investigated the differences between the metabolic profiles of HPV16-positive cervical SCC or precancerous lesions and those of HPV-negative normal controls using high-resolution magic angle spinning nuclear magnetic resonance (HRMAS ^1^H NMR). Based on the metabolic data, we subsequently detected the expression of several rate-limiting enzymes belonging to distinct metabolic pathways in cervical cancer and precancerous lesions by quantitative reverse transcription polymerase chain reaction (RT-qPCR) and immunohistochemistry.

## MATERIALS AND METHODS

### Samples

All procedures in the current study were performed following the Helsinki Declaration of 1975 (revised in 2000). Approval was obtained from the Ethics Committee of Xinjiang Medical University, China, with approval no. 20160226-08. Written informed consent was obtained from each participant. Analysis of all the data was conducted anonymously.

Patients who were diagnosed with cervical carcinoma or precancerous lesions in the Department of Gynecology at the People’s General Hospital of Xinjiang Uyghur Autonomous Region, Urumqi, China, between February 2015 and March 2016, were recruited. Pathologically, the patients were classified as patients with cervical SCC, CIN patients, and healthy controls (negative control [NC]) in accordance with the diagnostic criteria established by the World Health Organization and the Chinese Medical Association [20]. Patients with primary hypertension, coronary heart disease, bronchial asthma, diabetes mellitus, other complex metabolic diseases, and chronic inflammatory or infectious diseases were excluded from the study. Smokers were also excluded. In total, 52 fresh tissue specimens were collected from the participants through surgery. The pathological classification of the specimens revealed cervical SCC in 21 patients, CIN stages II-III in 20 patients, and 11 healthy controls. Following resection, the samples were immediately stored at −80°C. The patients had a median age of 45.2 years (ranging from 25 years to 69 years), and 36 of them had undergone childbirth. The NC group was matched by age and childbirth status to the patient group.

In addition, 123 cervical specimens (paraffin-embedded, 3-µm thickness) from the specimen bank at the Pathology Department of the hospital were used for immunohistochemical (IHC) staining. Two pathology experts were responsible for specimen selection; neither was aware of the purpose of this study. Among the specimens, 34 were from SCC patients, 38 were in CIN stage II-III, and 51 were normal.

The samples were clinically staged according to the literature [21]. Considering that most CIN stage I (CIN I) lesions can self-regress, whereas CIN II-III is normally considered significantly precancerous [20], CIN I specimens were excluded from this study.

### DNA extraction and HPV detection

The procedures were performed according to the literature [6]. Multifluorescent PCR assays were performed to determine the genotypes of HR HPVs, including HPV16, 18, 45, 31, 33, 52, 58, and 67, with a Real Quality RQ-HPV HR kit (Padua, Italy). The kit contained primers specific to HPV16, HPV18/45, and HPV31 and primers for HPV33/45/52/58/67. The amplification conditions began with an initial denaturation at 95°C for 1 min followed by 40 cycles of 95°C for 15 sec, 57°C for 30 sec, and 72°C for 30 sec. Human β-actin was used as the internal reference.

### Spectroscopic measurement of tissue specimens by HRMAS ^1^H NMR

The samples were collected within 30 min after resection and maintained at −80°C until analysis of the tissue riboflavin levels. The frozen tissue (15–25 mg) was packed in aluminum foil and pulverized by grinding under liquid nitrogen. HRMAS ^1^H NMR spectra were obtained with an NMR spectrometer (Varian Unity Inova600) under a Carr–Purcell–Meiboom–Gill (CPMG) pulse sequence (relaxation delay-90°-[τ-180°-τ]-acquire). For each specimen, 32,768 data points were yielded from 128 scans, the spectral width was 10,000 Hz, and the acquisition time was 2 sec. For assignment purposes, some samples underwent a two-dimensional NMR test, including 1H-1H homonuclear correlation spectroscopy, total correlation spectroscopy, and *J*-resolved spectroscopy [22].

### Data analysis

The chemical shifts of the tissue specimens were referenced to the anomeric proton of a-glucose at d5.233 ppm prior to data reduction into 2,834 integrated regions of 0.003 ppm corresponding to δH = 9.0–0.5 ppm [22]. Then, the reduced datasets were analyzed with Microsoft Excel. The regions corresponding to δH = 5.21–4.66 ppm were excluded due to the high variability in the water intensity [22]. The spectral data were input into SIMCA-P+ 11 software (Umetrics Inc., Umea, Sweden) for pattern recognition analysis. The concentrated NMR data were subjected to orthogonal projection to latent structure with discriminant analysis (OPLS-DA) with unit variance scaling [6]. R2Y and Q2 were used to describe the quality of the OPLS-DA. The coefficient outcomes revealed the variables that significantly contributed to the classification.

### RNA extraction and RT-qPCR

RNA extraction and RT-qPCR were performed according to the literature [22]. The primers for the target genes were synthesized by Takara Biotechnology Co., Ltd., Dalian, China. Each complementary DNA (cDNA) sample (20 ng) was reverse transcribed in a reaction volume of 25 µl. The amplification conditions were as follows: 95°C for 30 sec, followed by 40 cycles of 95°C for 5 sec and 60°C for 30 sec, and a final hold at 4°C for 10 min. The 2^−ΔΔCq^ method was utilized for quantitation of the expression of the target genes [23], with β-actin as the internal reference. The experiment was repeated three times for each sample.

### IHC staining

IHC staining was carried out according to the literature [22], and the procedures were performed in strict accordance with the instructions of the kits. Briefly, the antibodies included streptavidin peroxidase-conjugated primary rabbit polyclonal antibodies (Zhongshan Golden Bridge Biotech., Beijing, China) and biotin-labeled goat anti-rabbit secondary antibody (OriGene Technol, Beijing, China). A DAB kit (Zhongshan Golden Bridge Biotech) was used to visualize the staining. For evaluation, the samples were scored by two pathology experts, and the scale of the scores ranged from 0 to 3 [24]. The scores were given based on the following criteria: 0, no positively stained cells; 1, 0–30% positively stained cells; 2, >30 but <60% positively stained cells; and 3, >60% positively stained cells, which corresponded to negative, weak, moderate, and high signal intensities, respectively [22]. A consensus score between the two investigators was reached for each tissue slice. An overall score was then calculated by adding the two scores, and the overall scores were classified according to the following categories: 0–2, negative or weak expression; 3–4, moderate expression; and 5–6, strong expression.

### Statistical analysis

All the data are presented as the means ± standard deviations and were analyzed with SPSS Statistics for Windows, Version 17.0 (SPSS Inc., Chicago, IL, USA). All tests were two-tailed, and *p* < 0.05 indicated a significant difference. One-way ANOVA followed by a Dunnett’s *post hoc* test was performed for comparisons between and within groups. The Mann-Whitney U-test was used for the analysis of the scoring data obtained from IHC.

## RESULTS

### Profiling of tissue metabolites associated with cervical carcinogenesis

HPV infection was detected in 39 of the 52 fresh tissue specimens analyzed (Table 1). Specifically, HPV16 infection or co-infection with HPV16 and other HR HPV types was detected in 21 cases of SCC, 20 cases of CIN II-III, and one NC.

**TABLE 1:**
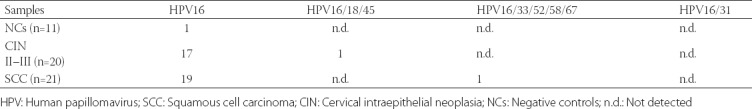
HPV genotyping of cervical lesions

**FIGURE 1:**
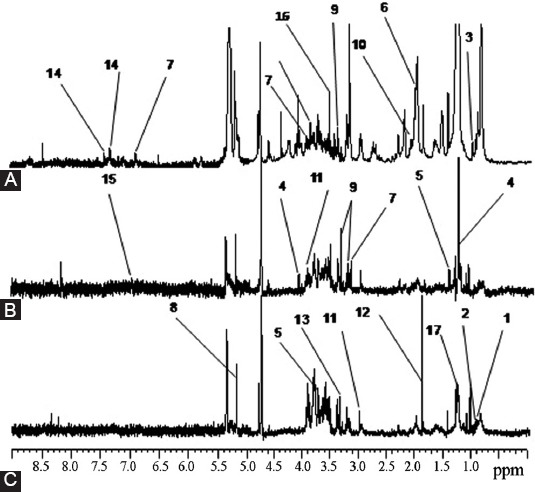
Average 600-MHz high-resolution magic angle spinning nuclear magnetic resonance (HRMAS ^1^H NMR) spectra of (A) squamous cell carcinoma (SCC) tumors, (B) cervical intraepithelial neoplasia (CIN) lesions, and (C) negative control (NC). Only the following significant metabolites are labeled in the three tissue metabolic profiles: 1, isoleucine; 2, leucine; 3, valine; 4, lactate; 5, alanine; 6, glycoprotein; 7, tyrosine; 8, α-glucose; 9, β-glucose; 10, methionine; 11, creatine; 12, acetate; 13, scyllo-inositol; 14, phenylalanine; 15, methylproline; 16, glycine; and 17, low-density lipoprotein (LDL).

**FIGURE 2:**
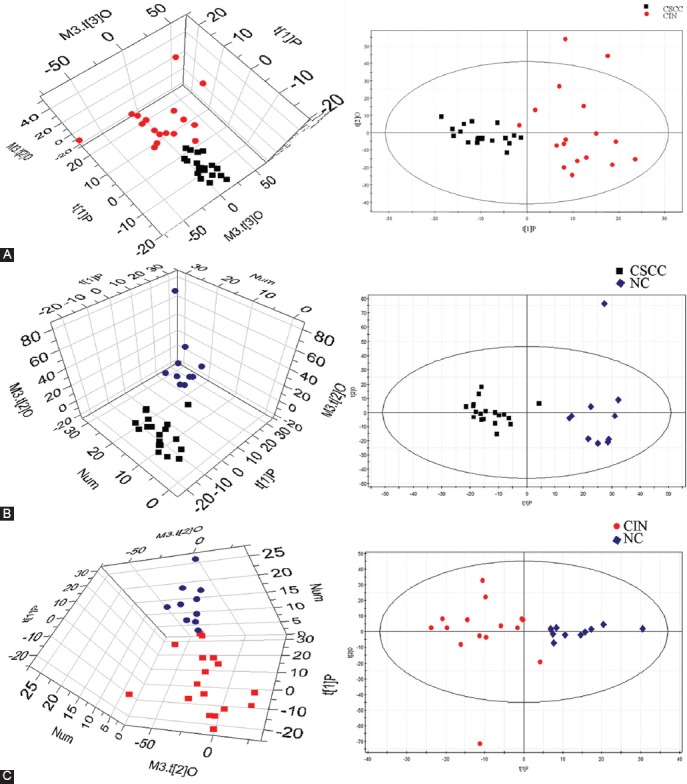
High-resolution magic angle spinning nuclear magnetic resonance (HRMAS ^1^H NMR) -based orthogonal projection to latent structure with discriminant analysis (OPLS-DA) score plots obtained from comparisons between (A) cervical squamous cell carcinoma (CSCC) and cervical intraepithelial neoplasia (CIN), (B) CSCC and negative control (NC), and (C) CIN and NC. CSCC (■), CIN (•), and NC (♦). The model parameters are as follows: R_2_X = 0.443, R_2_Y = 0.676, Q_2_ = 0.651, R_2_X = 0.34, R_2_Y = 0.87, Q_2_ = 0.84, R_2_X = 0.33, R_2_Y = 0.88, and Q_2_ = 0.93.

Subsequently, intact tissue specimens of HPV-positive cervical SCC (CSCC) and CIN II-III were analyzed using HRMAS ^1^H NMR. High-quality spectra were obtained from the 33 analyzed specimens, which included 16 CSCC and 17 CIN samples that were positive for HPV16 infection and 10 HPV-negative NCs. In total, 17 metabolites were identified within the range of 7.80–0.50 ppm based on the HRMAS ^1^H NMR spectra from all cervical tissue samples, and a visual inspection of all 1D CPMG spectra revealed significant differences among CSCC, CIN II-III, and NC groups (Figure 1). The identified metabolites showed well-defined peaks with no overlap in the 1D CPMG spectra and thus met the criteria for further quantification. For metabolic profiling, the mean-centered HRMAS ^1^H NMR data from all samples were subjected to OPLS-DA (Figure 2). The results showed intergroup metabolic differences between CSCC and NC, between CIN and NC, and between CSCC and CIN, which indicated that these three tissue types can be characterized by inherently different metabolic signatures.

The correlation coefficients for the 17 identified metabolites were calculated using the OPLS-DA model (Table 2), in which positive and negative signs represented decreases and increases in the given metabolites, respectively. Compared with CIN and NC, CSCC group showed significant increases in low-density lipoprotein (LDL), lactate, and alanine and decreases in α- and β-glucose, tyrosine, and phenylalanine. Compared with NC, CSCC group had decreased levels of isoleucine, methylproline, creatine, acetate, and scyllo-inositol. Notably, increased glycolysis might also be a signature of CIN, which are considered precursor lesions of cervical carcinoma, because the α- and β-glucose levels were decreased in CIN compared with NC group. These data and the findings obtained in previous studies suggest that enhanced glycolytic activity in tumor tissues may be accompanied by a deregulation of lipid and amino acid metabolism during cervical carcinogenesis.

**TABLE 2:**
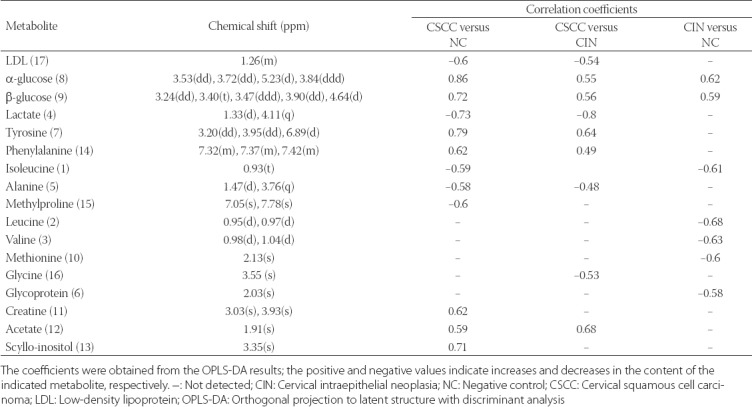
Correlation coefficients for metabolites showing significant differences among CSCC, CIN, and NC

### Aberrant regulation of key enzymes involved in metabolic pathways

**FIGURE 3:**
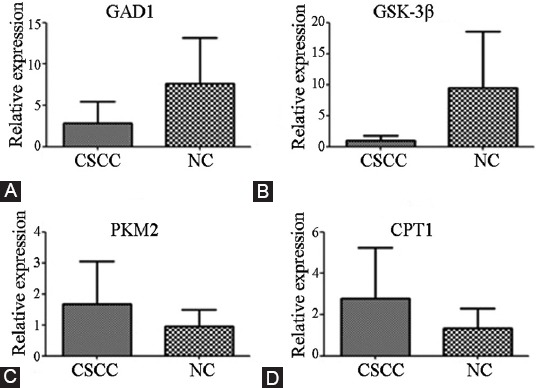
Differential expression of mRNAs encoding key enzymes between cervical squamous cell carcinoma (CSCC) and negative control (NC). (A), (B), (C), and (D) refer to glutamate decarboxylase 1 (*GAD1*), glycogen synthase kinase 3 beta (*GSK3B*), pyruvate kinase muscle isozyme 2 (PKM2), and carnitine palmitoyltransferase 1 (*CPT1*), respectively.

To further understand the mechanisms related to the aberrant regulation of metabolites in distinct metabolic pathways, the expression of four rate-limiting enzymes in sugar and lipid metabolism, namely, glycogen synthase kinase 3 beta (GSK3β) [25,26], the dimeric pyruvate kinase muscle isozyme 2 (PKM2) [27], carnitine palmitoyltransferase 1A (CPT1A) [28] and glutamate decarboxylase 1 (GAD1), was examined in the tumor and NC tissues. First, the transcription levels of these proteins in the 52 RNA samples were analyzed by RT-qPCR using specific primers for mRNAs encoding CPT1A, PKM2, and GSK3β (Table 3). The results showed that GSK3β and GAD1 were significantly downregulated and CPT1A and PKM2 were upregulated in CSCC compared with NC samples (*p* < 0.05), indicating that the catabolism of sugar and lipids was enhanced in tumor cells (Table 4 and Figure 3).

**TABLE 3:**
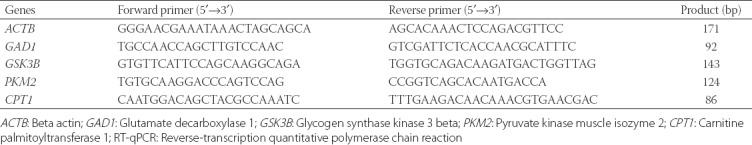
List of primers used in RT-qPCR analysis

**TABLE 4:**
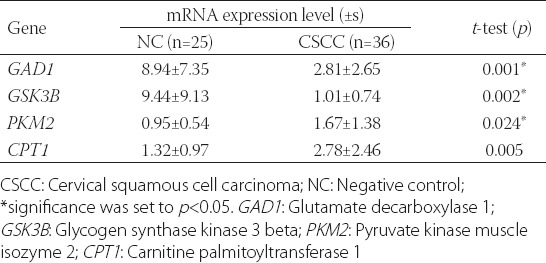
Altered expression of mRNAs encoding key enzymes in cervical lesions

According to HRMAS ^1^H NMR, high positivity for HPVs, particularly HPV16, was observed in SCC and CIN II-III groups, whereas HPV negativity was observed in NC group (Table 2), which agrees with our previous reports [29,30]. These results suggest that the HPV detection data are likely not suitable for the analysis of the correlation between HPV16 infection and gene expression. However, the high contrast obtained between HPV16 positivity in cervical lesions and HPV negativity in normal control tissue indicates that the regulation of gene expression dependent on HPV16 infection was likely integrated into the RT-qPCR results (Table 4).

**FIGURE 4:**
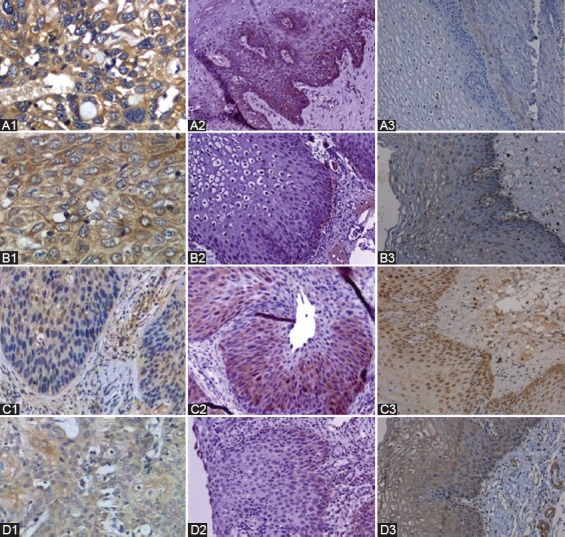
Immunohistochemical analysis. Panels A1 to D1 show the expression of pyruvate kinase muscle isozyme 2 (PKM2), carnitine palmitoyltransferase 1 (CPT1), glycogen synthase kinase 3 beta (GSK3β), and glutamate decarboxylase 1 (GAD1), respectively in the normal cervical epithelium. Panels A2 to D2 show the expression of PKM2, CPT1, GSK3β, and GAD1, respectively in CIN. Panels A3 to D3 show the expression of PKM2, CPT1, GSK3β, and GAD1, respectively in cervical carcinoma (original magnification, 200×).

IHC staining showed positive staining for all three proteins in the cytoplasm (Figure 4). The expression of GSK3β and GAD1 was significantly decreased in CSCC and CIN II-III compared with NC group [*p* < 0.05] (Table 5). The expression of PKM2 and CPT1A was significantly increased in CSCC and CIN compared with NC group (*p* < 0.05). Furthermore, the expression levels of these metabolites were significantly higher in CIN II-III than in CSCC group (Table 5).

**TABLE 5:**
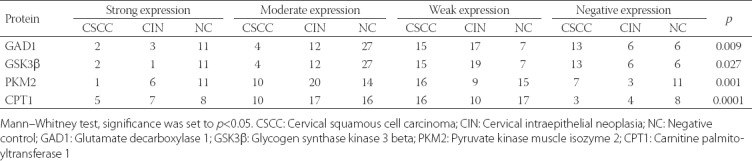
GAD1, GSK3β, PKM2, and CPT1 protein expression in cervical lesions

## DISCUSSION

Metabolites are the end products of gene expression and cellular regulatory processes. Tumorigenesis may modify global metabolism in the human body because different metabolic profiles in biofluids (plasma, urine, and other fluids) have been detected in human cancers. However, these changes are not fully understood in cervical carcinoma, particularly HPV-induced carcinogenesis.

This study identified a profile of 17 small molecular metabolites that showed differential expression in HPV16-positive SCC or its precursor lesions (CIN II-III) compared with its expression in HPV-negative NCs. Among the identified metabolites, the decrease in α- and β-glucose and the increase in lactate indicated a typical Warburg effect during cervical carcinogenesis [11,12]. Consistent with previous reports on blood-based metabolomics, we found an increase in LDL and alterations in multiple amino acids in cervical cancer tissues that might reflect an active lipid metabolism and a dual role of amino acid metabolism during tumor development [29]. Notably, tumor growth might drive the use of free amino acids in the blood circulation to facilitate rapid protein synthesis and altered energy metabolism in cervical cancer tissues [30]. This hypothesis is supported by our data and previous studies, which showed altered amino acid compositions in other tumor tissues [31]. High LDL levels in plasma and tumor tissues may be a signature characteristic for patients with cervical carcinoma and precancerous lesions [32]. Unsaturated lipids and triglycerides are elevated in intact tissues of cervical carcinoma during tumorigenesis or the transition from noninvasive to invasive cervical cancer [33]. In contrast, reduced levels of lipids/triglycerides have been detected in tumor tissues of other cancer types [34,35]. Notably, enhanced aerobic glycolysis has often been described as the signature of metabolic modifications in most solid tumors. This is consistent with the findings obtained in this study but does not agree with the results of a previous investigation of intact tumor tissues of cervical carcinoma [35,36]. The results of the current study and previous findings suggest that enhanced glycolytic activity in tumor tissues may be accompanied by the deregulation of lipid and amino acid metabolism during HPV-induced cervical carcinogenesis.

To further evaluate the impact of metabolic pathways on metabolic modification in cervical cancer, we determined the mRNA transcription and protein expression of key enzymes related to sugar and lipid metabolism, namely, GSK3β, PKM2 and CPT1A. The data demonstrated significant downregulation of GSK3β and upregulation of PKM2 and CPT1A in cervical carcinoma and precancerous lesions compared with NCs. Of these enzymes, GSK3 is a key enzyme that negatively regulates insulin-mediated glycogen synthesis and glucose homeostasis [25]. GSK3 has two isoforms with extensive homology, i.e., GSK3α and GSK3β, but most studies on GSK3 substrate diversity and physiological functions focused on GSK3β [26]. The function of GSK3β is tightly regulated by its phosphorylation state, i.e. this enzyme is active with phosphotyrosine (GSK3β^tyr216^) and inactive with phosphoserine (GSK3β^ser9^) [37]. Decreased GSK3β^tyr216^ and increased GSK3β^ser9^ levels have been reported in cervical cancer tissues, which indicates that activated GSK3β may play some roles in the inhibition of tumorigenesis [38]. The enhancer of zeste homolog 2 (EZH2) is elevated in cervical cancer tissues and activates Wnt/β-catenin signaling via the epigenetic silencing of GSK3β and p53 in cancer cells [39]. The tripartite motif-containing 29 (TRIM29) is also increased in cervical cancer lesions, and its downregulation results in enhanced GSK3β expression and inhibition of the epithelial-mesenchymal transition (EMT) in cancer cells [40], which could explain how GSK3β is inactivated by Wnt/β-catenin signaling to promote tumor progression [25,41]. Furthermore, GSK3β is recognized as the principal kinase that negatively regulates glycogen synthase in muscle cells [42]. GSK3β inactivation might result in decreased phosphorylation of kinesin motor proteins, which mediate the translocation of glucose transporters and increase glucose uptake [43]. These findings suggest that decreased expression of GSK3β may enable cervical cancer cells to exhibit enhanced glycolytic activity.

PKM2 is identical to PKM1 in length but shows differences in a 22-amino-acid peptide region due to alternative splicing [44]. PKM1 is found in most adult tissues, whereas PKM2 expression is enhanced in embryonic and regenerative tissues, particularly tumor tissues, where it plays an important role in the maintenance of aerobic glycolysis in tumor cells [45]. In cancer cells, PKM2 mainly exists as an inactive monomer and a less active dimer, but low levels of its active tetramer have also been detected [27]. The low-activity dimer maintains smooth aerobic glycolysis and promotes cell proliferation [46]. In contrast, the formation of tetrameric PKM2 through allosteric regulation might promote oxidative phosphorylation and thereby suppress tumorigenesis [47]. PKM2 activity is also regulated by phosphorylation; in particular, phosphorylation at Tyr105 disturbs the binding of PKM2 to its allosteric activator fructose-1,6-bisphosphate (FBP), thereby inhibiting tetramer formation and promoting aerobic glycolysis as a metabolic advantage to tumor cells [48]. Intriguingly, the HPV16 E7 oncoprotein might induce the tyrosine phosphorylation of PKM2 and promote neoplastic transformation [49]. In accordance with our metabolic data, the decrease in phenylalanine or increase in alanine also contributes to enhanced aerobic glycolysis in tumor cells because these amino acids might act as allosteric inhibitors to the tetramer conformations of PKM1 or PKM2 [50]. PKM2 elevation might contribute to radiation resistance or poor prognosis in patients with cervical carcinoma and oral SCC, whereas reduced PKM2 expression is associated with the inhibition of cell proliferation and EMT progression or enhanced radiation sensitivity in cancer cells [51-53]. These findings indicate that the detected increase in PKM2 in cervical cancer and precancerous lesions is likely associated with enhanced aerobic glycolysis and tumor growth.

Among different CPT1 isoforms, CPT1A is widely distributed, particularly in the liver, pancreas, kidney, brain, blood and embryonic tissues, and it serves as a key enzyme of lipid metabolism by facilitating the mitochondrial import of long-chain fatty acids for β-oxidation [54]. CPT1A exists as a trimer and hexamer (a dimer of trimers) under native conditions [55]. Hexameric CPT1A might form hetero-oligomeric complexes with other enzymes and mitochondrial channel proteins, such as long-chain acyl-CoA synthetase (ACSL) and voltage-dependent anion channel (VDAC), to facilitate the transfer of fatty acids into mitochondria [56]. Consistent with our results, CPT1A expression is increased in tumor specimens of pancreatic ductal adenocarcinoma and ovarian cancer and is associated with chemotherapy resistance or poor survival, whereas CPT1A knockdown induces energy stress, cell-cycle arrest, and sensitivity to glucose deprivation in cancer cells or inhibits xenograft growth [57,58]. Alternative splicing of the same gene might produce two CPT1A isoforms, termed CPT1Av1 and CPT1Av2, which show differences in 17 amino acids at the C-terminal region. The shorter form, CPT1Av2, has a high affinity for histone deacetylase 1 (HDAC1) and may be involved in cancer survival and invasion [28]. Therefore, the observed increase in expression of CPT1A in cervical carcinoma and precancerous lesions indicates the involvement of CPT1A in lipid metabolism modifications and tumor progression.

GAD1 is a rate-limiting enzyme in the production of γ-aminobutyric acid (GABA) and found in the GABAergic neurons in the central nervous system. In contrast to previous findings that reported an elevated GAD1 expression in oral, nasopharyngeal, and gastric cancers [59,60], we detected a significant decrease in GAD1 expression in cervical cancer tissues. The impaired GAD1 expression may be related to the gene hypermethylation in some neoplastic tissues [61]. However, the role of GAD1 in carcinogenesis is largely unknown, because most of the studies focus on its impact on neuronal functions.

Due to the preliminary nature and dependence on clinical diagnoses, this study suffers from some limitations, including the small sample size obtained based on the validation requirements, the insufficient number of HPV-negative samples from cervical carcinoma and precursor lesions, and the lack of *in vitro* cellular experiments that could further reveal the mechanisms underlying metabolic regulation in cervical carcinoma.

## CONCLUSION

In the present study, we obtained a profile of 17 metabolites that show differential expression in HPV16-positive cervical carcinoma or precancerous lesions compared with HPV-negative controls. In particular, the decrease in glucose, increase in lactate, and alterations in multiple amino acids indicate that a switch to enhanced glycolysis accompanied by a distortion of lipid and amino acid metabolism occurs during HPV-induced carcinogenesis. This conclusion is further supported by analyses of the expression of GAD1, GSK3β, PKM2, and CPT1 in cervical lesions, and the results of these analyses imply that enhanced aerobic glycolysis and disturbed lipid metabolism are advantageous to tumor growth.

## References

[1] Torre LA, Bray F, Siegel RL, Ferlay J, Lortet-Tieulent J, Jemal A (2015). Global cancer statistics, 2012. CA Cancer J Clin.

[2] Koh WJ, Greer BE, Abu-Rustum NR, Apte SM, Campos SM, Cho KR (2015). Cervical cancer, version 2.2015. J Natl Compr Canc Netw.

[3] Denny L (2012). Cervical cancer:Prevention and treatment. Discov Med.

[4] Echelman D, Feldman S (2012). Management of cervical precancers:A global perspective. Hematol Oncol Clin North Am.

[5] Kim MA, Oh JK, Kim BW, Chay D, Park DC, Kim SM (2012). Prevalence and seroprevalence of low-risk human papillomavirus in korean women. J Korean Med Sci.

[6] Tulake W, Yuemaier R, Sheng L, Ru M, Lidifu D, Abudula A (2018). Upregulation of stem cell markers ALDH1A1 and OCT4 as potential biomarkers for the early detection of cervical carcinoma. Oncol Lett.

[7] Ferenczy A, Franco E (2002). Persistent human papillomavirus infection and cervical neoplasia. Lancet Oncol.

[8] Yu LL, Chen W, Lei XQ, Qin Y, Wu ZN, Pan QJ (2016). Evaluation of p16/Ki-67 dual staining in detection of cervical precancer and cancers:A multicenter study in China. Oncotarget.

[9] Petry KU, Schmidt D, Scherbring S, Luyten A, Reinecke-Lüthge A, Bergeron C (2011). Triaging pap cytology negative, HPV positive cervical cancer screening results with p16/Ki-67 dual-stained cytology. Gynecol Oncol.

[10] Wentzensen N, Schiffman M, Palmer T, Arbyn M (2016). Triage of HPV positive women in cervical cancer screening. J Clin Virol.

[11] Asgari Y, Zabihinpour Z, Salehzadeh-Yazdi A, Schreiber F, Masoudi-Nejad A (2015). Alterations in cancer cell metabolism:The Warburg effect and metabolic adaptation. Genomics.

[12] Yonezawa K, Nishiumi S, Kitamoto-Matsuda J, Fujita T, Morimoto K, Yamashita D (2013). Serum and tissue metabolomics of head and neck cancer. Cancer Genomics Proteomics.

[13] Battini S, Faitot F, Imperiale A, Cicek AE, Heimburger C, Averous G (2017). Metabolomics approaches in pancreatic adenocarcinoma:Tumor metabolism profiling predicts clinical outcome of patients. BMC Med.

[14] Budczies J, Brockmöller SF, Müller BM, Barupal DK, Richter-Ehrenstein C, Kleine-Tebbe A (2013). Comparative metabolomics of estrogen receptor positive and estrogen receptor negative breast cancer:Alterations in glutamine and beta-alanine metabolism. J Proteomics.

[15] Tian Y, Xu T, Huang J, Zhang L, Xu S, Xiong B (2016). Tissue metabonomic phenotyping for diagnosis and prognosis of human colorectal cancer. Sci Rep.

[16] Teng CF, Hsieh WC, Yang CW, Su HM, Tsai TF, Sung WC (2016). A biphasic response pattern of lipid metabolomics in the stage progression of hepatitis B virus X tumorigenesis. Mol Carcinog.

[17] Sah RG, Sharma U, Parshad R, Seenu V, Mathur SR, Jagannathan NR (2012). Association of estrogen receptor, progesterone receptor, and human epidermal growth factor receptor 2 status with total choline concentration and tumor volume in breast cancer patients:An MRI and in vivo proton MRS study. Magn Reson Med.

[18] Grinde MT, Skrbo N, Moestue SA, Rødland EA, Borgan E, Kristian A (2014). Interplay of choline metabolites and genes in patient-derived breast cancer xenografts. Breast Cancer Res.

[19] Costello LC, Feng P, Milon B, Tan M, Franklin RB (2004). Role of zinc in the pathogenesis and treatment of prostate cancer:Critical issues to resolve. Prostate Cancer Prostatic Dis.

[20] Saslow D, Runowicz CD, Solomon D, Moscicki AB, Smith RA, Eyre HJ (2002). American Cancer Society guideline for the early detection of cervical neoplasia and cancer. CA Cancer J Clin.

[21] Pecorelli S, Benedet JL, Creasman WT, Shepherd JH (1999). FIGO staging of gynecologic cancer 1994-1997 FIGO Committee on Gynecologic Oncology International Federation of Gynecology and Obstetrics. Int J Gynaecol Obstet.

[22] Hasim A, Ma H, Mamtimin B, Abudula A, Niyaz M, Zhang LW (2012). Revealing the metabonomic variation of EC using ¹H-NMR spectroscopy and its association with the clinicopathological characteristics. Mol Biol Rep.

[23] Livak KJ, Schmittgen TD (2001). Analysis of relative gene expression data using real-time quantitative PCR and the 2(-delta delta C(T)) method. Methods.

[24] Mason DY, Immunocytochemical analysis of human tissue (1992). Oxford Textbook of Pathology.

[25] Frame S, Cohen P (2001). GSK3 takes centre stage more than 20 years after its discovery. Biochem J.

[26] Rayasam GV, Tulasi VK, Sodhi R, Davis JA, Ray A (2009). Glycogen synthase kinase 3:More than a namesake. Br J Pharmacol.

[27] Wong N, Ojo D, Yan J, Tang D (2015). PKM2 contributes to cancer metabolism. Cancer Lett.

[28] Pucci S, Zonetti MJ, Fisco T, Polidoro C, Bocchinfuso G, Palleschi A (2016). Carnitine palmitoyl transferase-1A (CPT1A):A new tumor specific target in human breast cancer. Oncotarget.

[29] Hasim A, Ali M, Mamtimin B, Ma JQ, Li QZ, Abudula A (2012). Metabonomic signature analysis of cervical carcinoma and precancerous lesions in women by (1)H NMR spectroscopy. Exp Ther Med.

[30] Ye N, Liu C, Shi P (2015). Metabolomics analysis of cervical cancer, cervical intraepithelial neoplasia and chronic cervicitis by 1H NMR spectroscopy. Eur J Gynaecol Oncol.

[31] Tripathi P, Somashekar BS, Ponnusamy M, Gursky A, Dailey S, Kunju P (2013). HR-MAS NMR tissue metabolomic signatures cross-validated by mass spectrometry distinguish bladder cancer from benign disease. J Proteome Res.

[32] Ye N, Liu C, Shi P (2015). Metabolomics analysis of cervical cancer, cervical intraepithelial neoplasia and chronic cervicitis by 1H NMR spectroscopy. Eur J Gynaecol Oncol.

[33] De Silva SS, Payne GS, Thomas V, Carter PG, Ind TE, deSouza NM (2009). Investigation of metabolite changes in the transition from pre-invasive to invasive cervical cancer measured using (1)H and (31)P magic angle spinning MRS of intact tissue. NMR Biomed.

[34] Wang AS, Lodi A, Rivera LB, Izquierdo-Garcia JL, Firpo MA, Mulvihill SJ (2014). HR-MAS MRS of the pancreas reveals reduced lipid and elevated lactate and taurine associated with early pancreatic cancer. NMR Biomed.

[35] Rocha CM, Barros AS, Gil AM, Goodfellow BJ, Humpfer E, Spraul M (2010). Metabolic profiling of human lung cancer tissue by 1H high resolution magic angle spinning (HRMAS) NMR spectroscopy. J Proteome Res.

[36] Torregrossa L, Shintu L, Nambiath Chandran J, Tintaru A, Ugolini C, Magalhães A (2012). Toward the reliable diagnosis of indeterminate thyroid lesions:A HRMAS NMR-based metabolomics case of study. J Proteome Res.

[37] Medina M, Wandosell F (2011). Deconstructing GSK-3:The fine regulation of its activity. Int J Alzheimers Dis.

[38] Rath G, Jawanjal P, Salhan S, Nalliah M, Dhawan I (2015). Clinical significance of inactivated glycogen synthase kinase 3βin HPV-associated cervical cancer:Relationship with Wnt/β-catenin pathway activation. Am J Reprod Immunol.

[39] Chen Q, Zheng PS, Yang WT (2016). EZH2-mediated repression of GSK-3βand TP53 promotes Wnt/β-catenin signaling-dependent cell expansion in cervical carcinoma. Oncotarget.

[40] Xu R, Hu J, Zhang T, Jiang C, Wang HY (2016). TRIM29 overexpression is associated with poor prognosis and promotes tumor progression by activating Wnt/β-catenin pathway in cervical cancer. Oncotarget.

[41] Chen RH, Ding WV, McCormick F (2000). Wnt signaling to beta-catenin involves two interactive components Glycogen synthase kinase-3beta inhibition and activation of protein kinase C. J Biol Chem.

[42] McManus EJ, Sakamoto K, Armit LJ, Ronaldson L, Shpiro N, Marquez R (2005). Role that phosphorylation of GSK3 plays in insulin and Wnt signalling defined by knockin analysis. EMBO J.

[43] Morfini G, Szebenyi G, Elluru R, Ratner N, Brady ST (2002). Glycogen synthase kinase 3 phosphorylates kinesin light chains and negatively regulates kinesin-based motility. EMBO J.

[44] Noguchi T, Inoue H, Tanaka T (1986). The M1-and M2-type isozymes of rat pyruvate kinase are produced from the same gene by alternative RNA splicing. J Biol Chem.

[45] Dayton TL, Jacks T, Vander Heiden MG (2016). PKM2, cancer metabolism, and the road ahead. EMBO Rep.

[46] Mazurek S (2011). Pyruvate kinase type M2:A key regulator of the metabolic budget system in tumor cells. Int J Biochem Cell Biol.

[47] Anastasiou D, Yu Y, Israelsen WJ, Jiang JK, Boxer MB, Hong BS (2012). Pyruvate kinase M2 activators promote tetramer formation and suppress tumorigenesis. Nat Chem Biol.

[48] Hitosugi T, Kang S, Vander Heiden MG, Chung TW, Elf S, Lythgoe K (2009). Tyrosine phosphorylation inhibits PKM2 to promote the Warburg effect and tumor growth. Sci Signal.

[49] Zwerschke W, Mazurek S, Massimi P, Banks L, Eigenbrodt E, Jansen-Dürr P (1999). Modulation of type M2 pyruvate kinase activity by the human papillomavirus type 16 E7 oncoprotein. Proc Natl Acad Sci U S A.

[50] Morgan HP, O'Reilly FJ, Wear MA, O'Neill JR, Fothergill-Gilmore LA, Hupp T (2013). M2 pyruvate kinase provides a mechanism for nutrient sensing and regulation of cell proliferation. Proc Natl Acad Sci U S A.

[51] Zhao Y, Shen L, Chen X, Qian Y, Zhou Q, Wang Y (2015). High expression of PKM2 as a poor prognosis indicator is associated with radiation resistance in cervical cancer. Histol Histopathol.

[52] Cheng KY, Hao M (2017). Mammalian target of rapamycin (mTOR) regulates transforming growth factor-β1 (TGF-β1)-induced epithelial-mesenchymal transition via decreased pyruvate kinase M2 (PKM2) expression in cervical cancer cells. Med Sci Monit.

[53] Shen Y, Chen M, Huang S, Zou X (2016). Pantoprazole inhibits human gastric adenocarcinoma SGC-7901 cells by downregulating the expression of pyruvate kinase M2. Oncol Lett.

[54] López-Viñas E, Bentebibel A, Gurunathan C, Morillas M, de Arriaga D, Serra D (2007). Definition by functional and structural analysis of two malonyl-coA sites in carnitine palmitoyltransferase 1A. J Biol Chem.

[55] Faye A, Esnous C, Price NT, Onfray MA, Girard J, Prip-Buus C (2007). Rat liver carnitine palmitoyltransferase 1 forms an oligomeric complex within the outer mitochondrial membrane. J Biol Chem.

[56] Lee K, Kerner J, Hoppel CL (2011). Mitochondrial carnitine palmitoyltransferase 1a (CPT1a) is part of an outer membrane fatty acid transfer complex. J Biol Chem.

[57] Luo J, Hong Y, Tao X, Wei X, Zhang L, Li Q (2016). An indispensable role of CPT-1a to survive cancer cells during energy stress through rewiring cancer metabolism. Tumour Biol.

[58] Shao H, Mohamed EM, Xu GG, Waters M, Jing K, Ma Y (2016). Carnitine palmitoyltransferase 1A functions to repress FoxO transcription factors to allow cell cycle progression in ovarian cancer. Oncotarget.

[59] Kimura R, Kasamatsu A, Koyama T, Fukumoto C, Kouzu Y, Higo M (2013). Glutamate acid decarboxylase 1 promotes metastasis of human oral cancer by β-catenin translocation and MMP7 activation. BMC Cancer.

[60] Lee YY, Chao TB, Sheu MJ, Tian YF, Chen TJ, Lee SW (2016). Glutamate decarboxylase 1 overexpression as a poor prognostic factor in patients with nasopharyngeal carcinoma. J Cancer.

[61] Yan H, Tang G, Wang H, Hao L, He T, Sun X (2016). DNA methylation reactivates GAD1 expression in cancer by preventing CTCF-mediated polycomb repressive complex 2 recruitment. Oncogene.

